# Clinical Characteristics and Symptomatology Associated With Dengue Fever

**DOI:** 10.7759/cureus.26677

**Published:** 2022-07-09

**Authors:** Hareem Arshad, Mahvish Bashir, Uzema S Mushtaq, Hafsa Imtiaz, Rahimeen Rajpar, Muhammad Fiyaz Alam, Saher Fatima, Anjum Rehman, Kiran Abbas, Abdul Subhan Talpur

**Affiliations:** 1 Department of Medicine, Jinnah Sindh Medical University, Karachi, PAK; 2 Department of Medicine, Jinnah Postgraduate Medical Centre, Karachi, PAK; 3 Department of Nephrology, Urology, Dialysis & Renal Transplantation, Bahawal Victoria Hospital, Bahawalpur, PAK; 4 Department of Nephrology, Quaid-e-Azam Medical College, Bahawalpur, PAK; 5 Department of Obstetrics and Gynecology, Shaheed Mohtarma Benazir Bhutto Medical College, Karachi, PAK; 6 Department of Medicine, Liaquat University of Medical and Health Sciences, Jamshoro, PAK

**Keywords:** immunoglobulin m, immunoglobulin g, dengue, infection, fever, dengue serotypes, complication

## Abstract

Background

Early diagnosis and prompt treatment are critical to reducing overall morbidity and mortality associated with dengue fever. Thus, to better understand the condition, the present study was conducted to assess the clinical signs and symptomatology associated with dengue fever in patients in a tertiary care hospital.

Methods

This prospective observational study was conducted at a tertiary care hospital in Karachi, Pakistan between July and December 2021. All patients who tested positive for the dengue virus either based on antigen or antibodies were included in the study. Convenient sampling was used. A structured proforma was used for data collection. Microsoft Excel (Microsoft Corporation, Redmond, WA) and Statistical Package for the Social Sciences (SPSS, IBM Corp., Armonk, NY) were used for the entry and analysis of data, respectively.

Results

More than half of the patients were suffering from fever (82.5%), headache/body ache/joint pain (80.5%), and vomiting (55%). Bleeding was observed in 16 (8%) patients and was directly related to platelet count (OR: 0.981; 95% CI: 0.971-0.992), and more than half of the patients (56%) required platelet transfusion. Laboratory values included a mean platelet count of 145.22 ± 90.36 thousand, a mean total leukocyte count (TLC) of 6.87 ± 5.76 thousand, and a mean hemoglobin level of 13.71 ± 2.11 g/dl. Of the patients, 171 (85.5%) individuals tested positive for antigen nonstructural protein 1 (Ns1Ag), and 68 (34%) tested positive for either immunoglobulin G (IgG) or immunoglobulin M (IgM), or both dengue-specific antibodies. Those with dengue-specific antibodies were less likely to bleed as 6.2% were IgG and IgM positive and 31.2% were positive for both antibodies. The regression model showed a significant relationship between bleeding and platelet transfusion (p < 0.001), hospital stay (p < 0.005), and diarrhea (p < 0.001).

Conclusion

In conclusion, the study revealed that males were more frequently infected with the virus as compared to females. Furthermore, fever, headache/joint pain/body aches, diarrhea, and low platelet count are the major clinical and laboratory outcomes. Patients with a low level of platelets are more prone to bleeding, and platelet transfusion increased survival chances in such patients.

## Introduction

Dengue fever is a vector-borne viral infection caused by a virus of the genus *Flavivirus*, which belongs to the family *Flaviviridae*, consisting of single-stranded RNA [1]. Dengue is endemic in the tropical and subtropical areas of the world with 100 billion dengue cases being reported worldwide and approximately 50-200 million cases with 500,000 incidences of dengue hemorrhagic fever and over 20,000 deaths documented every year across the world [2].

Pakistan is a subtropical country and it is endemic for vector-borne diseases such as dengue and malaria. The incidence of dengue has been expanding in Pakistan and yearly morbidity and mortality have been on the rise. Dengue was first introduced in Pakistan at the Karachi seaport [1,3]. The first case was reported in Karachi in 1994, and a severe outbreak was reported in 1995 in Hub, Balochistan [4]. In Pakistan, four dengue serotypes circulate the entire year, with peak outbreaks occurring in post-monsoon months. In Pakistan, it has become a significant threat since 2005, putting millions of residents at risk [4]. Contributing factors to the expansion of the dengue virus include increased population growth rate, global warming, unplanned urbanization, inefficient mosquito control, frequent air travel, and lack of healthcare facilities [5].

Clinical manifestations may vary from being asymptomatic [6], or with fever, myalgias, and rash to dreaded complications, such as shock and hemorrhagic fever [7]. Roughly, about 20% of infections are asymptomatic, with individuals suffering from disease outcomes, which include non-severe to mildly severe to severe outcomes of the broad clinical spectrum [8]. Recently, some studies have reported atypical presentation of dengue. Pothapregada et al. revealed some atypical manifestations of dengue fever in the sample population including lymphadenopathy, biphasic pyrexia, hepatitis, febrile diarrhea, refractory shock, altered consciousness, portal hypertension, cholecystitis, acute respiratory distress syndrome, myocarditis, and pericardial effusion [9].

Diagnosis relies on laboratory evaluation. Early diagnosis and prompt treatment are critical to reducing overall morbidity and mortality [10]. The treatment options include symptomatic management with hydration, analgesics, and control of complications, as currently there is no antiviral therapy available and the disease is usually self-limiting [11-13]. Our research aimed to study the overview of dengue fever and its clinical manifestations, both typical and atypical presentations, in the population of Karachi, Sindh, Pakistan.

## Materials and methods

A prospective observational study was conducted at a tertiary care hospital in Karachi, Pakistan between 1st July and 31st December 2021. The data collection commenced after ethical approval was obtained from the institutional review board (IRB) of Jinnah Postgraduate Medical Centre (reference number: F2-102-IRB-2021-GENL/5446/JPMC). A nonprobability convenience sampling technique was employed to enroll the participants.

The sample size was calculated using OpenEpi by using a confidence level of 99% and a margin of error of 5%. By keeping an expected proportion of diarrhea at 8.1% [12], a sample size of 198 was calculated. Considering a dropout rate of approximately 10%, a total sample size of 218 was obtained.

**Figure 1:**
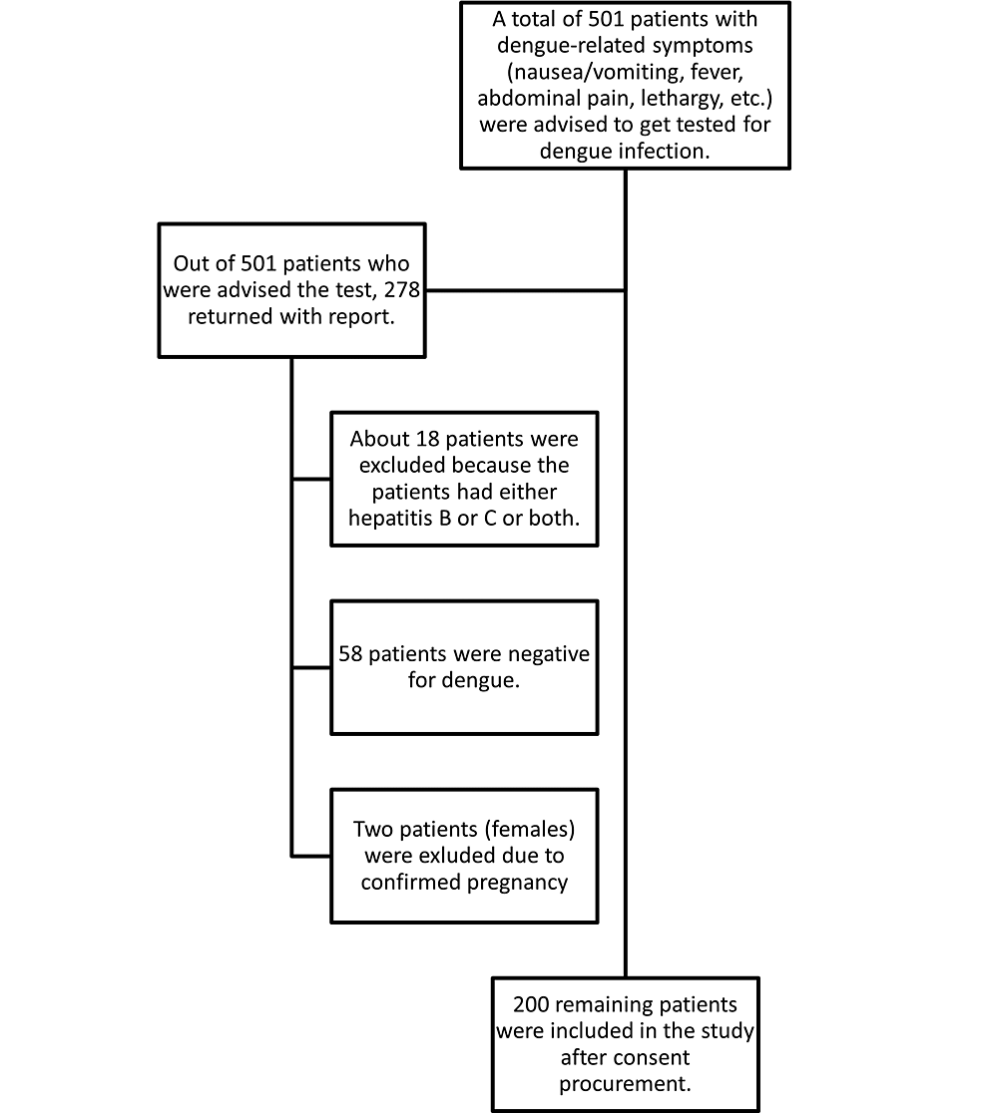
Flowchart illustrating the sample selection and patient distribution

All patients, irrespective of gender and age, who presented to the outpatient department during the study period and were positive for dengue infection based on either nonstructural protein 1 (Ns1Ag) antigen or dengue virus-specific antibodies (immunoglobulin G (IgG) and immunoglobulin M (IgM)) were included in the study. Patients with unconfirmed diagnosis and co-infection with hepatitis B or C were excluded from the study. Pregnant women were also not included (Figure 1).

Informed verbal and written consent was obtained from the participants after the goal of the study was narrated to them. The participants were ensured of complete anonymity and confidentiality of the data. A pre-structured proforma was used for data collection. Clinical symptoms and laboratory values including the hematological parameters of all patients were gathered.

The data were entered into Microsoft Excel (Microsoft Corporation, Redmond, WA). Analysis of the data was done using the Statistical Package for the Social Sciences (SPSS) version 26.0 (IBM Corp., Armonk, NY). All continuous values, including hemoglobin (Hb), platelet count, and other red blood cell indices, were presented as mean and standard deviation. All categorical variables were presented as frequency and percentage. Multivariate regression was applied to adjust the impact of clinical and laboratory variables on the dengue symptomatology. A p-value of <0.05 was set as the cut-off for statistical significance.

## Results

A total of 200 patients suffering from dengue were included in this study, out of which 136 (68%) were males. Among these, 171 (85.5%) tested positive for antigen Ns1Ag. Upon testing for detection of antibodies, nine (4.5%) tested positive for IgG antibodies, 30 (15%) tested positive for IgM antibodies, and 29 (14.5%) tested positive for both IgG and IgM antibodies. The overall prevalence of antibodies specific to dengue, i.e., IgG, IgM, and both of these, was 34%. The mean age of the patients was 37.75 ± 14.47 years. Descriptive statistics of laboratory values of participants are given in Table 1.

**Table 1:** Descriptive statistics of laboratory parameters (n = 200)

Parameter	Mean (SD)	Minimum	Maximum
Hemoglobin (mg/dl)	13.72 (±2.11)	6	20
Platelets count (x1,000)	145.23 (±90.36)	2.37	758.6
Total leukocytes count (x1,000)	6.86 (±5.76)	1.69	70
Hematocrit (%)	41.20 (±6.10)	16.1	93
Alanine transaminase (U/L)	72.5 (±199.86)	10	2,560
Urea (grams)	36.36 (±25.43)	10	176
Creatinine (mg/dl)	0.96 (±0.77)	0.24	7.95

**Figure 2:**
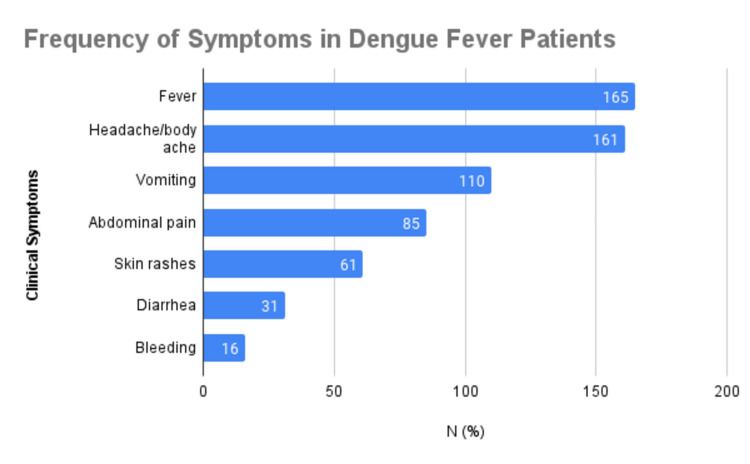
Frequency of clinical symptoms observed among patients

The clinical symptoms of the patients enrolled in the study are summarized in Figure 2. More than half of the patients were suffering from fever (165, 82.5%), headache/body ache/joint pain (161, 80.5%), and vomiting (110, 55%). Abdominal pain was reported by 85 (42.5%), skin rashes by 61 (30.5%), and diarrhea by 31 (15.5%) patients. Only 16 patients (8%) had bleeding. Comorbidities were observed in 42 (21%) of total patients. The minimum stay observed was zero days, and the maximum was 10 days, with a mean stay of 3.01 ± 1.86 days.

Table 2 shows the β-value of the regression equation and the level of significance. By reviewing the p-value for the predictor of bleeding, it was found that platelet count had a statistically significant impact on bleeding in dengue patients. No bleeding was used as a reference category. With each level of increase in platelet count, the level of bleeding will decrease by 0.981.

**Table 2:** Univariate analysis using logistic regression for bleeding in dengue patients

Dependent variable: bleeding (yes)			
	Odds ratio	95% CI	P-value
Platelets count (x1,000)	0.98	0.97-0.99	0.000
Pseudo R^2^	0.21		
Observations			

Variables are used to build a multivariate logistic model to predict bleeding in patients with dengue. The British Medical Association (BMA) package was used to select the optimal model with two criteria to evaluate model quality: post prob and Bayesian Information Criterion (BIC). Four best models were proposed by the package. All four models showed two variables, i.e., platelet transfusion and diarrhea, and models one to three had additional variables, i.e., hospital stay, platelet count, and headache/body ache/joint pain, respectively. One of these models was chosen because it had the highest post prob (90%) and the lowest BIC (-1002.00).

The overall survival rate in case of bleeding was 93.75%, while in patients with no bleeding, it was observed to be 98.36%. In the multivariable logistic regression, we adjusted for the hospital stay, platelet transfusion, and diarrhea. We found a significant association of these variables with the presence of bleeding in dengue patients. Patients with each additional stay at the hospital were more likely to bleed (OR: 1.02; 95% CI: 1.01-1.04). Patients with diarrhea symptoms were significantly associated with increased occurrence of bleeding in dengue patients (OR: 1.35; 95% CI: 1.25-1.46). Moreover, adjusted models further confirm the significant association of positive platelet transfusion with the presence of bleeding (OR: 1.60; 95% CI: 1.43-1.78) (Table 3).

**Table 3:** Multivariable analysis using logistic regression for bleeding in the study sample

Dependent variable: bleeding (yes)			
Predictors	Adjusted odds ratio	95% CI	P-value
Hospital stay (days)	1.02	1.01-1.04	0.002
Platelet transfusion			
Negative	1		
Positive	1.60	1.43-1.78	0.000
Diarrhea			
Negative	1		
Positive	1.35	1.25-1.46	0.000
Pseudo R^2^	0.52		
Observations	200		

Among 200 total dengue patients, 16 patients had bleeding as an outcome. Table 4 shows the data of participants in whom bleeding occurred and also those in whom no bleeding occurred concerning clinical symptoms and antigen/antibody detection. Out of these 16 patients, most were Ns1Ag positive (93.75%), had a fever (81.25%), headache/body ache/joint pain (68.75%), and diarrhea (81.25%), while those with dengue-specific antibodies were less likely to bleed, as 6.2% were IgG and IgM positive and 31.2% were positive for both antibodies. During the study period, four patients expired.

**Table 4:** Summary descriptive table by groups of “bleeding”

Parameters	Bleeding		
Antigen/antibody	Yes (%)	No (%)	P-value
Nonstructural protein 1 (Ns1Ag)			0.329
Positive	15 (93.8)	156 (84.4)	
Negative	1 (6.2)	28 (15.2)	
Immunoglobulin G (IgG)			0.725
Positive	1 (6.2)	8 (4.3)	
Negative	15 (93.8)	176 (95.7)	
Immunoglobulin M (IgM)			0.307
Positive	1 (6.2)	29 (15.8)	
Negative	15 (93.8)	155 (84.2)	
IgG and IgM			0.047
Positive	5 (31.2)	24 (13.0)	
Negative	11 (68.8)	160 (87.0)	
Fever			0.891
Yes	13 (81.2)	152 (82.6)	
No	3 (18.8)	32 (37.2)	
Headache/body ache/joint pain			0.216
Yes	11 (68.8)	150 (81.5)	
No	5 (31.2)	34 (18.5)	
Vomiting			0.346
Yes	7 (43.8)	103 (56.0)	
No	9 (56.2)	81 (44.0)	
Abdominal pain			0.343
Yes	5 (31.2)	80 (43.5)	
No	11 (68.8)	104 (56.5)	
Rash			0.618
Yes	4 (25.0)	57 (31.0)	
No	12 (75.0)	127 (69.0)	
Diarrhea			0.000
Yes	13 (81.2)	18 (9.8)	
No	3 (18.8)	166 (90.2)	
Outcome			0.206
Survive	15 (93.8)	181 (98.4)	
Expired	1 (6.2)	3 (1.6)	

## Discussion

Dengue fever is a frequently occurring viral disease that is associated with a significant burden on health care [14]. We reported the seroprevalence of the dengue virus in patients. The overall prevalence of antibodies specific to the dengue virus was 34%. Seroprevalence of these antibodies in our study is much less than that reported in the epidemiological study conducted in Khyber Pakhtunkhwa, where it was found in more than half of the study subjects (n = 319), and a study conducted in Lahore, where it was 88% (n = 245), including 48.7% IgM and 39.5% IgG antibodies. It was higher than a similar study conducted in Hyderabad, where the seroprevalence of IgM was 16.5% and IgG was 12.4% (n = 49) [15,16]. Similarly, the seroprevalence of dengue-based Ns1Ag was much higher (85.5%) than reported by a similar study in India, where it was 35.24% [17].

Based on gender, the results of seroprevalence observed were consistent with other studies in India, Singapore, and Pakistan, as males were tested positive more than females [18-20]. This may be attributed to differences in social and cultural lifestyles, as males are involved more in outdoor activities with less body cover. Most patients were symptomatic and symptoms significantly associated with dengue were fever, skin rash, headache, joint pain, vomiting, and diarrhea, which is consistent with the findings of the above studies [15].

Bleeding in patients infected with the dengue virus is multifactorial and is directly related to platelet count [21]. Platelet count, hospital stay, and diarrhea were found as significant risk factors leading to bleeding, while other laboratory values and clinical symptoms were not found to have a significant impact on bleeding. In our study, the occurrence of bleeding was lesser than that reported in another study, where bleeding occurred in 32.78% of patients with dengue fever and 54.05% of patients with dengue hemorrhagic fever. Platelet transfusion was significantly related to bleeding (OR 1.60; 95% CI: 1.43-1.78; p < 0.001) in dengue positive patients.

Shakya et al. revealed several atypical presentations in dengue patients including petechiae, subconjunctival hemorrhage, nosebleeds, as well as heavy menstrual bleeding in women. Furthermore, the study also observed that eight individuals suffered from acute kidney injury, and six patients presented with central nervous system dysfunction [22]. In a case series from India, patients presented with dengue encephalitis and viral pneumonia. The study revealed that one of the patients suffered from refractory shock and expired [23]. Thus, it can be seen that atypical manifestations in dengue patients are being reported in high numbers.

The survival rate in case of bleeding was 93.75%, while in patients with no bleeding, it was observed to be 98.36%, which is higher than that reported in Lahore, where it was 81% [24]. Currently, there is no active surveillance system in Pakistan for vector-borne diseases such as malaria and dengue and it relies on a passive system that does not conclude the entire population of the entire country, especially areas with poor health facilities. Inauguration of such an active system will aid the health department in monitoring such outbreaks more closely and identifying the areas with severe outbreaks. On other hand, there is no approved and specific treatment for dengue virus infection, so supportive symptomatic treatment is mandatory. A prompt diagnosis and control of the spread of the virus through vector mosquitoes is a standard approach to control the infection [25].

The present study had certain limitations. First, the study was conducted in a single center, and second, the sample size was rather small. Both these factors limit the interference of our findings to a larger population. Finally, the overall mortality rate and long-term outcomes of patients with dengue fever were not considered due to resource restrictions and a high rate of loss to follow-up of patients. A further large-scale study with a more diversified sample population is warranted.

## Conclusions

It was concluded from the study that most of the patients were carrying the antigen of the dengue virus, which can be transmitted to other persons. Males were more infected with the virus as compared to females. Moreover, fever, headache/joint pain/body aches, diarrhea, and low platelet count are the major clinical and laboratory outcomes, respectively. Patients with a low level of platelets are more prone to bleeding, and platelet transfusion increased survival chances in such patients. Furthermore, we found that patients with dengue-specific antibodies were less likely to bleed as 6.2% were IgG and IgM positive and 31.2% were positive for both antibodies.
